# Network Effects on Scientific Collaborations

**DOI:** 10.1371/journal.pone.0057546

**Published:** 2013-02-28

**Authors:** Shahadat Uddin, Liaquat Hossain, Kim Rasmussen

**Affiliations:** 1 Project Management Program and Centre for Complex Systems Research, The University of Sydney, Sydney, Australia; 2 Faculty of Engineering and IT, The University of Sydney, Sydney, Australia; Aalto University, Finland

## Abstract

**Background:**

The analysis of co-authorship network aims at exploring the impact of network structure on the outcome of scientific collaborations and research publications. However, little is known about what network properties are associated with authors who have increased number of joint publications and are being cited highly.

**Methodology/Principal Findings:**

Measures of social network analysis, for example network centrality and *tie strength*, have been utilized extensively in current co-authorship literature to explore different behavioural patterns of co-authorship networks. Using three SNA measures (i.e., *degree centrality*, *closeness centrality* and *betweenness centrality*), we explore scientific collaboration networks to understand factors influencing performance (i.e., citation count) and formation (*tie strength* between authors) of such networks. A *citation count* is the number of times an article is cited by other articles. We use co-authorship dataset of the research field of ‘*steel structure*’ for the year 2005 to 2009. To measure the strength of scientific collaboration between two authors, we consider the number of articles co-authored by them. In this study, we examine how *citation count* of a scientific publication is influenced by different centrality measures of its co-author(s) in a co-authorship network. We further analyze the impact of the network positions of authors on the strength of their scientific collaborations. We use both correlation and regression methods for data analysis leading to statistical validation. We identify that *citation count* of a research article is positively correlated with the *degree centrality* and *betweenness centrality* values of its co-author(s). Also, we reveal that *degree centrality* and *betweenness centrality* values of authors in a co-authorship network are positively correlated with the strength of their scientific collaborations.

**Conclusions/Significance:**

Authors’ network positions in co-authorship networks influence the performance (i.e., *citation count*) and formation (i.e., *tie strength*) of scientific collaborations.

## Introduction

Study of co-authorship network has been the subject of intense interest in recent years because this type of network not only depicts academic society but also represents the structure of our knowledge in an open innovation community [1]–[3]. Co-authorship network is an important class of social network. A social network is defined as a collection of individuals, each of whom is acquainted with some other subset of others by one or more different types of relations such as friendship, kinship and co-authorship [4]. Researchers have been analyzing co-authorship network extensively to explore factors affecting behaviour, performance and motivation of scientific collaborations [5]–[7]. Somewhat similar to the much studied citation networks, co-authorship implies a much stronger bond among authors than citation. Unlike citation networks where nodes are papers and the links between them are citations [8], in a co-authorship network nodes represent authors and links between nodes imply a scientific collaboration.

Co-authorships of research collaborations and publications have a long history. The first collaborative scientific paper was published in 1665 [9]. The first issue of the journal ‘*Philosophical Transactions of the Royal Society* (*Phil. Trans.*)’ was published on 6 March 1665. The Royal Society of London is the publisher of this journal and the first issue of this journal was edited by the society’s first secretary Hendry Oldenburg. This very first volume of this journal published many single-author papers (e.g., Petit [10] ) and few two-author papers (e.g., Moray and Du Son [11]). During the last few decades, the scientific collaboration has increased rapidly in diverse research areas [12]–[15] and researchers have been exploring research questions related to the outcome measures (e.g., citation count) of their scientific collaborations. Mazloumian [16] examined, for instance, the predictive capability of citation count and found that citation counts are reliable predictors of future success (e.g., future citation counts and attract research grant) for scientists. Landmark papers of famous scientists are not only acknowledged by many immediate citations but also they boost citation rates of the previous publications of the corresponding scientist [17]. The analysis of co-authorship networks for exploring patterns of scientific collaboration is a comparatively young research discipline. During the 1990s, a number of authors pointed out the potential utility of co-authorship data and in some cases performed small-scale statistical analyses [18]–[20]. An early example of the analysis of co-authorship network is the *Erdös Number Project* [21]. Paul Erdös was an influential but itinerant Hungarian mathematician. He was one of the most prolific authors of research papers and had been involved in writing at least 1401 papers, which was more than the number of publications of any other mathematician who lived before or during his time. In bibliographical terms, the Erdös number represents a mathematician’s proximity to the great man.

Co-authorship data have attracted considerable interest in recent years because co-authorship data are the source of the largest (free and computerized as well) social networks available among researchers [22], [23]. Researchers have approached the analysis of co-authorship data in various ways such as basic level statistical analysis using charts and regression [24], and structure and pattern of co-authorship networks [5], [25]. Liu et al. [26] adopted the social network measures of *degree*, *closeness*, *betweenness* and *eigenvector* centrality to explore individuals’ positions in a given co-authorship network. Yan and Ding [27] later utilized basic centrality measures to explore, at an actor-level, how network positions of authors in a co-authorship network affect the citation counts of their papers. In their research, they consider that authors of a paper share the same *citation count* (i.e., *citation count* of that paper) regardless of the order of authors in the author list of that paper. Like them, we also use basic social network centrality measures in this study. However, their works were author-centric. They explored the effect of the network position of an author on the *citation count* of all her/his papers. On the other hand, our works are paper-centric. We investigate the effect of the network positions of all co-authors of a research paper on its *citation count*.

We have two research objectives in this study. First, we aim to explore how *citation count* of a scientific paper is influenced by the network positions of its co-author(s) in a co-authorship network. Second, we explore how authors’ network positions influence their strength of relations with others in a co-authorship network. The outcomes of these two research objectives can contribute significantly to the state of the art in co-authorship network studies. Scientists would be able to know the impact of their network positions in the co-authorship network on the citation counts of their published papers and on the strength of their scientific relations with their colleagues. Researchers would be able to identify potential researchers in their own research areas. In order to establish research collaborations, this information might be very helpful for early career researchers and those who wish to establish external research collaborations. Not only that, a virtual ranking of all authors of any research area could be developed from the information of their network positions in co-authorship networks. Therefore, outcomes of this study would help in identifying potential researchers and in developing effective and efficient research collaborations. The following two questions motivate this study:

1. How is the citation count of a scientific paper influenced by the network positions of its co-author(s) in a co-authorship network?
2. How is the strength of scientific relations (i.e., co-authorship relations) between two authors influenced by their network positions in a co-authorship network?

We use the terms paper, research publication, research article, research paper and journal article interchangeably. Similarly, the words researcher, author and scientist are exchangeable in this paper. Node, actor and individual are also interchangeable. The rest of this paper is organized as follows. In section two, we illustrate the conceptualization of our two research questions. This is followed by the research methodology as described in section three. In section four, we posit the research findings of this study. Finally, in section five we make a general discussion about the research findings of this study. In this section, we also posit the conclusive remarks of this study.

## Methods

### Conceptualization of Research Questions

In this research, we study co-authorship networks to explore what network attributes of authors in a co-authorship network influence the citation counts of scientific papers and the strength of relations with the other members of that co-authorship network. More specifically, if a paper has two co-authors (say *Au1* and *Au2*) who are also part of a co-authorship network having five authors, then this study examines: (i) which network attributes of *Au1* and *Au2* affect the *citation count* of that paper; and (ii) what network attributes of *Au1* and *Au2* affect their strength of relation with the remaining authors (i.e., three authors) of that co-authorship network.

**Figure 1:**
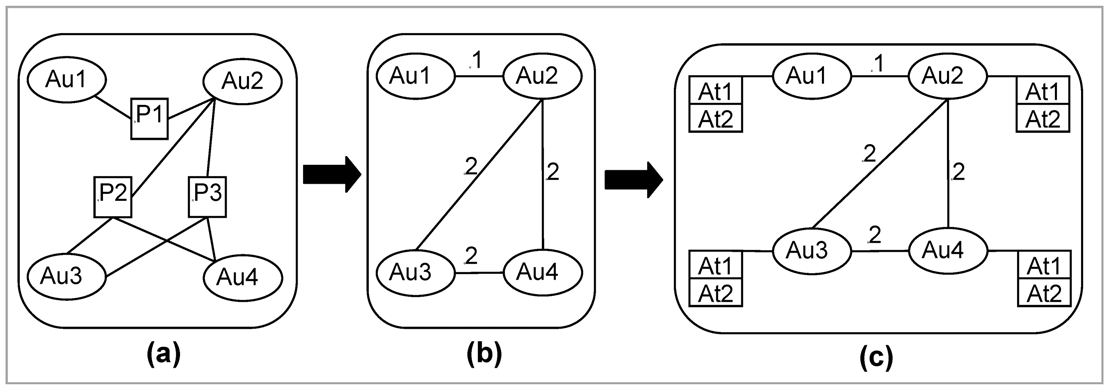
Illustration of a co-authorship network. (a) *author-paper* network for three papers written by four authors; (b) corresponding co-authorship network; and (c) co-authorship network showing same network attributes for each author. *Au* stands for *Author*, *P* stands for *Paper* and *At* stands for network attribute (e.g., *degree centrality*) of authors. Although in this figure we consider two attributes (i.e., *At1* and *At2*) for illustration, we consider network attributes of *degree centrality*, *closeness centrality* and *betweenness centrality* of all authors for research analysis in this study.

**Figure 2:**
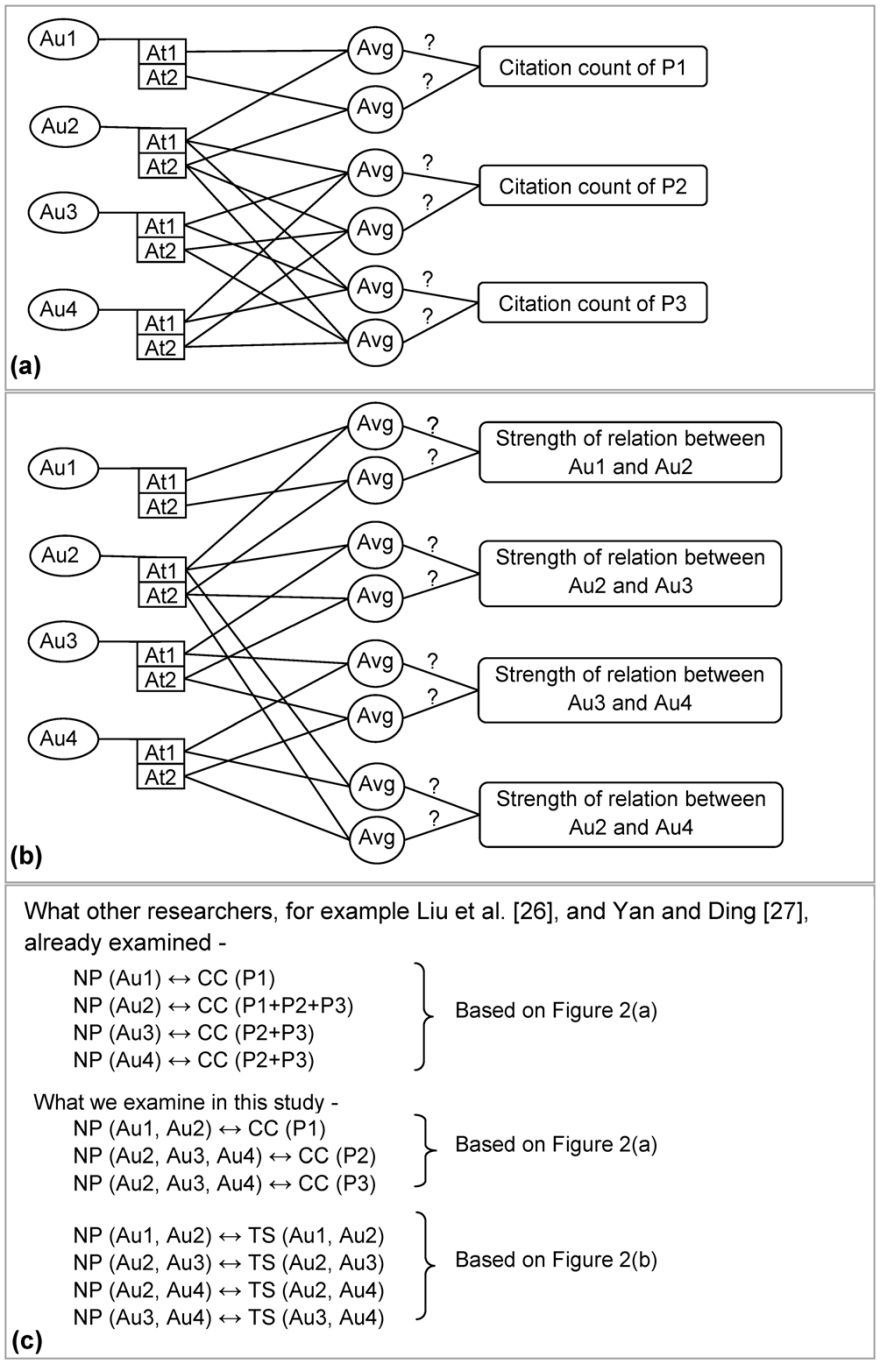
Conceptualization of the research questions of this study. (a) Illustration of the first research question (i.e., how the *citation count* of a scientific paper is affected by the network positions of its all co-author(s) in a co-authorship network?) based on Figure 1C. *Avg* stands for statistical function *Average* which is used to normalize different network attributes (i.e., *degree centrality*, *closeness centrality* and *betweenness centrality*) of authors. The “?” symbol above the line indicates, whether or not, the measure on its left hand side has any impact on the measure on its right hand side. (b) Illustration of the second research question (i.e., how the strength of scientific relations (i.e., co-authorship relations) between two authors is affected by their network positions in a co-authorship network?) based on Figure 1C. *Avg* and “?” represent the same as like in (a). (c) Summary of research investigations. *NP* stands for *Network Position* in respect of network measures considered in this study (i.e., *degree centrality*, *closeness centrality* and *betweenness centrality*), *CC* stands for *Citation Count* and *TS* stands for *Tie strength*. The symbol ‘↔’ stands, whether the left hand measure of the symbol has any impact on its right hand measure.

Figure 1 and Figure 2 conceptualize our research questions with illustration. Figure 1A shows *author-paper* network for three papers (i.e., *P1*, *P2* and *P3*) that are written by four authors (i.e., *Au1*, *Au2*, *Au3* and *Au4*). The corresponding co-authorship network of Figure 1A is illustrated in Figure 1B. In Figure 1C, we exhibit, in addition to co-authorship network, the network measures/attributes (i.e., *At1* and *At2*) of each co-author. These network measures for each co-author are measured from Figure 1B. We consider only two network measures for illustration. There could be more network measures for authors to be considered that depend mainly on the research question(s) under consideration. Figure 2A shows the illustration of our first research question (i.e., how is the *citation count* of a research paper influenced by the network measures of its co-author(s)?). Our second research question (i.e., how is the strength of scientific relations between two authors influenced by their network positions in a co-authorship network?) is illustrated in Figure 2B. These illustrations of our research questions (i.e., Figure 2A and Figure 2B) are based on Figure 1C. The summary of our research investigation is illustrated in Figure 2C.

### Data Source

In this study, we utilize co-authorship data from the research field of ‘*steel structure*’. We explore our research questions at two levels: (i) for the complete dataset; and (ii) for small groups within the complete dataset. For the group level, we choose two research groups from Monash University, Australia and National University of Singapore (NUS). These two groups have a very good reputation for their scientific contributions to the research field of ‘*steel structure*’. That means we consider three separated co-authorship networks – one for the complete research dataset and two (i.e., NUS and Monash University) for the group level dataset. Obviously, the group level dataset are part of the complete research dataset. Then we explore our two research questions for these three co-authorship networks separately. We consider research publications from the year 2005 to 2009. We extracted research publication details for our research dataset from Scopus, which is one of the largest abstract and citation databases for peer-reviewed literature and other scientific publications [28].

We first create a query to search research articles from Scopus. In this query, we specify ‘*steel structure’* as search phrase, and seek out this phrase in the title, keywords and abstract section of research articles. We also define the time frame (i.e., 2005 to 2009) and a list of journals to limit our search. The journal list, as named in Table 1, and the single search phrase (i.e., ‘*steel structure*’) were suggested by a domain expert of the ‘*steel structures’* research area. Then we import all journal articles in comma-separated value (CSV) format resulting from our query. In this imported dataset we notice that there are some journal articles which do not have complete bibliographic information such as author details, citation details and publication year. We do not consider those articles in the data analysis. By using affiliation information of authors, we then extract publication details for the ‘*steel structure*’ research groups of Monash University and National University of Singapore separately. Basic statistics of the research publications of these two groups are shown in Table 2.

**Table 1:** List of journals.

No.	Journal Name	No.	Journal Name
1	Advances in Structural Engineering	10	International Journal of Impact Engineering
2	Canadian Journal of Civil Engineering	11	Journal of Constructional Steel Research
3	Computers and Structures	12	Journal of Engineering Mechanics
4	Earthquake Engineering and Structural Dynamics	13	Journal of Structural Engineering
5	Engineering Fracture Mechanics	14	Journal of Structural Engineering New York, N.Y.
6	Engineering Structures	15	Steel and Composite Structures
7	Fatigue and Fracture of Engineering Materials and Structures	16	Structural Engineer
8	Fire Safety Journal	17	Structural Engineer London
9	Advances in Structural Engineering		

**Table 2:** Basic statistics of the co-authorship data used in this study.

Statistical Items	NUS	Monash University	Complete Dataset
Number of papers	39	56	888
Number of authors	36	57	1825
Average authors per paper	2.56	2.52	2.82
No. Of 1-author papers	5 (12.82%)	10 (17.86%)	81 (9.12%)
No. Of 2-author papers	12 (30.77%)	19 (33.93%)	378 (42.57%)
No. Of ≥3 author papers	22 (56.41%)	27 (48.21%)	429 (48.31%)
Average papers per author	1.08	0.98	0.49
No. Of authors having 1 paper	22 (61.11%)	34 (59.65%)	1470 (80.55%)
No. Of authors having 2 papers	8 (22.22%)	9 (15.79%)	224 (12.27%)
No. Of authors having ≥3 papers	6 (16.67%)	14 (24.56%)	131 (7.18%)
Average collaborators per authors	3.61	3.44	2.84
No. Of authors having 1 collaborator	5 (13.88%)	15 (26.32%)	383 (20.99%)
No. Of authors having 2 collaborators	13 (36.12%)	14 (24.56%)	598 (32.77%)
No. Of authors having ≥3 collaborators	18 (50.00%)	28 (49.12%)	844 (46.24%)

### Network Measures Used in this Study

Various network measures such as centrality, *tie strength* and *density* have gained significant interest in recent years [29], [30] and in many disciplines they play an important role to quantify and identify informal network which functions at level beyond the formal and traditional structure of relationships [31]–[33]. In this study, we use four network measures. Three of them are basic network centrality measures: (i) *degree centrality*; (ii) *closeness centrality*; and (iii) *betweenness centrality*. The fourth one is the *tie strength* measure, which was first introduced by Mark Granovetter [34].

The selection of these four network measures for analyzing co-authorship network is guided by three network theories: (i) Bavelas’ Centralization Theory [35]; (ii) Freeman’s Centrality Theory [36]; and (iii) Granovetter’s Strength of Weak Tie Theory [34]. Bavelas theory states that network structures of communication and collaboration among individuals have a positive impact on performance. Freeman’s centrality theory posits that centralities of actors in a network have an impact on their ability to perform. *Tie strength* among actors in a network has an impact on the ease of knowledge transfer and sharing, according to *Strength of Weak Tie Theory* of Granovetter.

*Degree centrality* or *degree*, which is defined by the number of direct links that a particular node has in a network [29], is one of the basic network centrality measures of social network analysis (SNA). It highlights highly connected nodes and, eventually, reflects those nodes having more direct contrast and adjacency with others in a given network [29]. As the co-authorship networks are, by definition, undirected, in this study we use simple degree centrality measure for authors. In a co-authorship network having *n* actors, the equation of *degree centrality* for an author *Au_i_* can be defined as follows [29]:
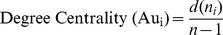
Where, 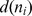 represents the number of authors with whom author *i* is connected in the co-authorship network.

*Closeness centrality* expands the definition of *degree centrality* by focusing on how close a node is to all other nodes of the network. For an individual node, it represents to what extent a node is in a close position to the remaining nodes of the network. In a co-authorship network having *n* authors, *closeness centrality* for an author *Au_i_* can be defined by the following equation [29]:
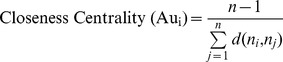
Where, 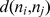 is the number of lines in the shortest distance between author *i* and author *j*, and the sum is taken over all *i≠j*.

*Betweenness centrality* is obtained by determining how often a particular node is found on the shortest path between any pair of nodes in the network. It views an actor as being in a favoured position to the extent that the actor falls on the shortest paths between other pairs of actors in the network. That is, nodes that occur on many shortest paths between other pair of nodes have higher *betweenness centrality* than those that do not [36]. The co-authorship networks considered in this research are connected. In a co-authorship network of size *n*, the *betweenness centrality* for an author *Au_i_* can be represented by the following equation [29]:
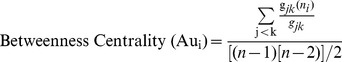
Where, *i ≠ j ≠ k;* 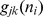 represents the number of shortest paths linking the two authors that contain author *i*; and 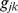 is the number of shortest paths linking author *j* and author *k*.

*Tie Strength* defines the quality of relationship between two actors in a network. According to Granovetter [34], the strength of relation between two actors can be expressed as a combination of the amount of time and the reciprocal services which characterize the tie between them. In the context of co-authorship network, *tie strength* represents the strength of relation between two scientists in terms of scientific collaborations, research outcomes, joint publications, and so on. In this study, we consider the total number of papers co-authored by two scientists in measuring the *tie strength* of their research collaboration.

### Approach of Research Analysis

**Figure 3:**
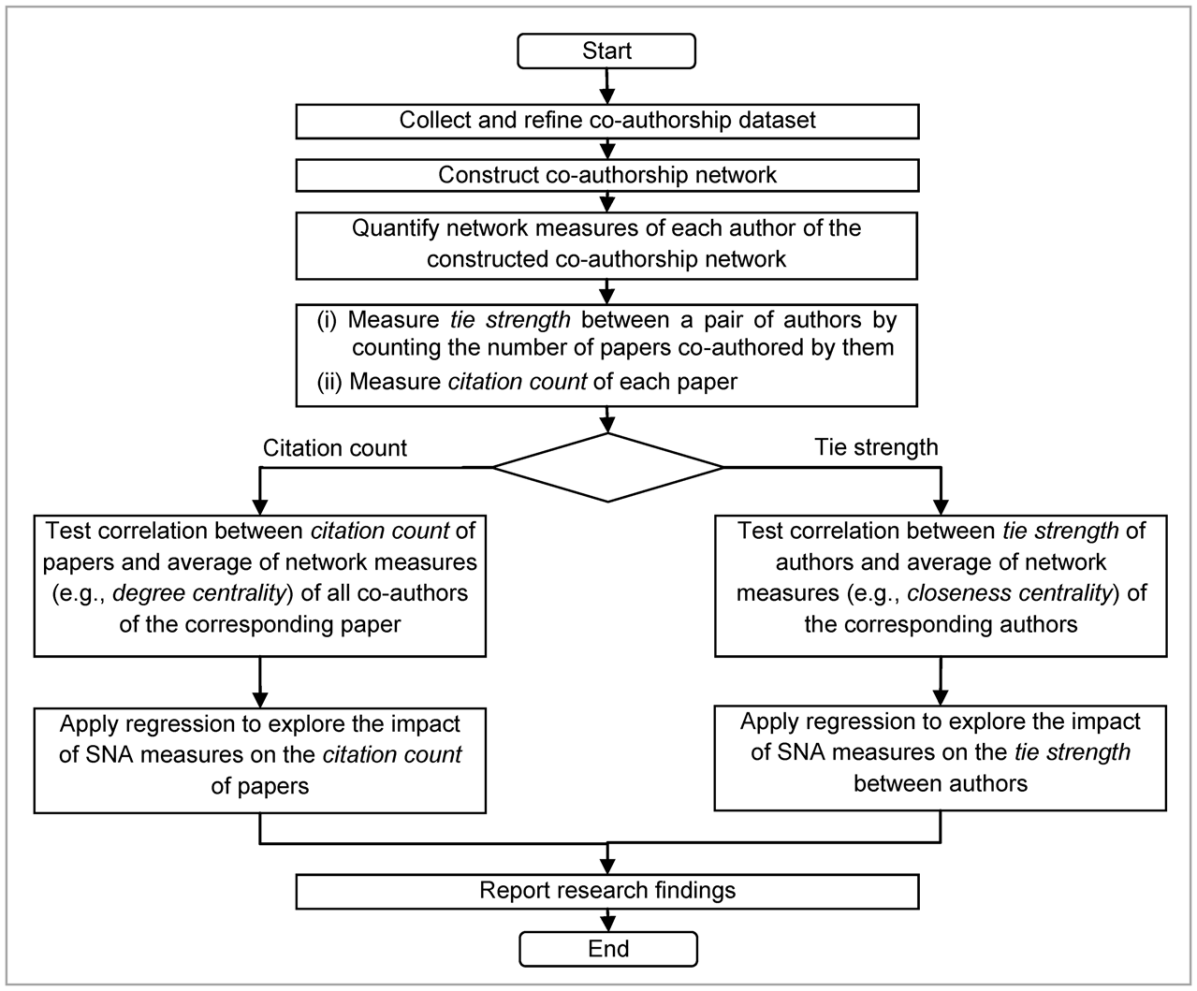
Research analysis process.

Using co-authorship dataset, we first construct co-authorship networks for the two research groups of NUS and Monash University. We then quantify network measures (i.e., *degree centrality*, *closeness centrality* and *betweenness centrality*) for each author of those co-authorship networks. We use ORA, which is a dynamic network analysis tool capable of performing node-level and network-level analyses of weighted networks [37], to measure these three network centrality values for each author. *Degree centrality* and *betweenness centrality* values of all co-authors are averaged respectively for each paper so that a single *degree* and *betweenness* value will be associated with each paper. For measuring *tie strength* between two authors, we consider the number of scientific papers co-authored by those two authors. Finally, we use the Spearman correlation test to check whether network measures of authors have any impact on citation counts, and on their strength of scientific collaborations. The Spearman correlation test approach is chosen because we notice that the distributions of all network measures considered in this research are non-normal. After that, we use the regression method to explore the impact of SNA measures on the *citation count* of papers and *tie strength* between authors. Figure 3 illustrates the flow chart of research analysis process followed in this study.

## Results

In this section, we discuss the findings of this study. We present these research findings under the following three subtitles.

### Impact of Network Positions of Co-authors on Citation Counts of Publications

The correlation coefficient values between each of three centrality measures and *citation count* are being presented in Table 3. For our complete research dataset, it is revealed that the average of the *degree centrality* and *betweenness centrality* values of all co-authors of a scientific publication have positive correlations with the citation count of that paper (rho = 0.397, p<0.01 at 2-tailed and 0.349, p<0.01 at 2-tailed respectively). For NUS and Monash University research group, we notice that the average of the *degree centrality* values of all co-authors of a scientific publication has a positive correlation with the *citation count* of that publication (rho = 0.326, p<0.05 at 2-tailed and rho = 0.433, p<0.05 at 2-tailed respectively). It is also evident that *betweenness centrality* shows a similar relationship with the *citation count* of a research publication for both NUS and Monash University research group (rho = 0.384 p<0.05 at 2-tailed and rho = 0.412, p<0.05 at 2-tailed respectively). However, *closeness centrality* does not show any significant correlation for the complete research dataset as well as for both for NUS and Monash University research group, with the *citation count* of a research paper.

**Table 3:** Correlation matrix between three network centrality measures and *citation count*.

Correlations				
		Citation Count		
Spearman’s rho		NUS	Monash University	Complete research dataset
Degree centrality	Correlation Coefficient	.326**	.433**	.397**
	Sig. (2-tailed)	.011	.002	.001
	N	39	56	888
Closeness centrality	Correlation Coefficient	.039	.191	.012
	Sig. (2-tailed)	.341	.209	.109
	N	39	56	888
Betweenness centrality	Correlation Coefficient	.384**	.456**	.349**
	Sig. (2-tailed)	.028	.027	.001
	N	39	56	888

** Correlation is significant at the 0.01 level (2-tailed).

* Correlation is significant at the 0.05 level (2-tailed).

**Figure 4:**
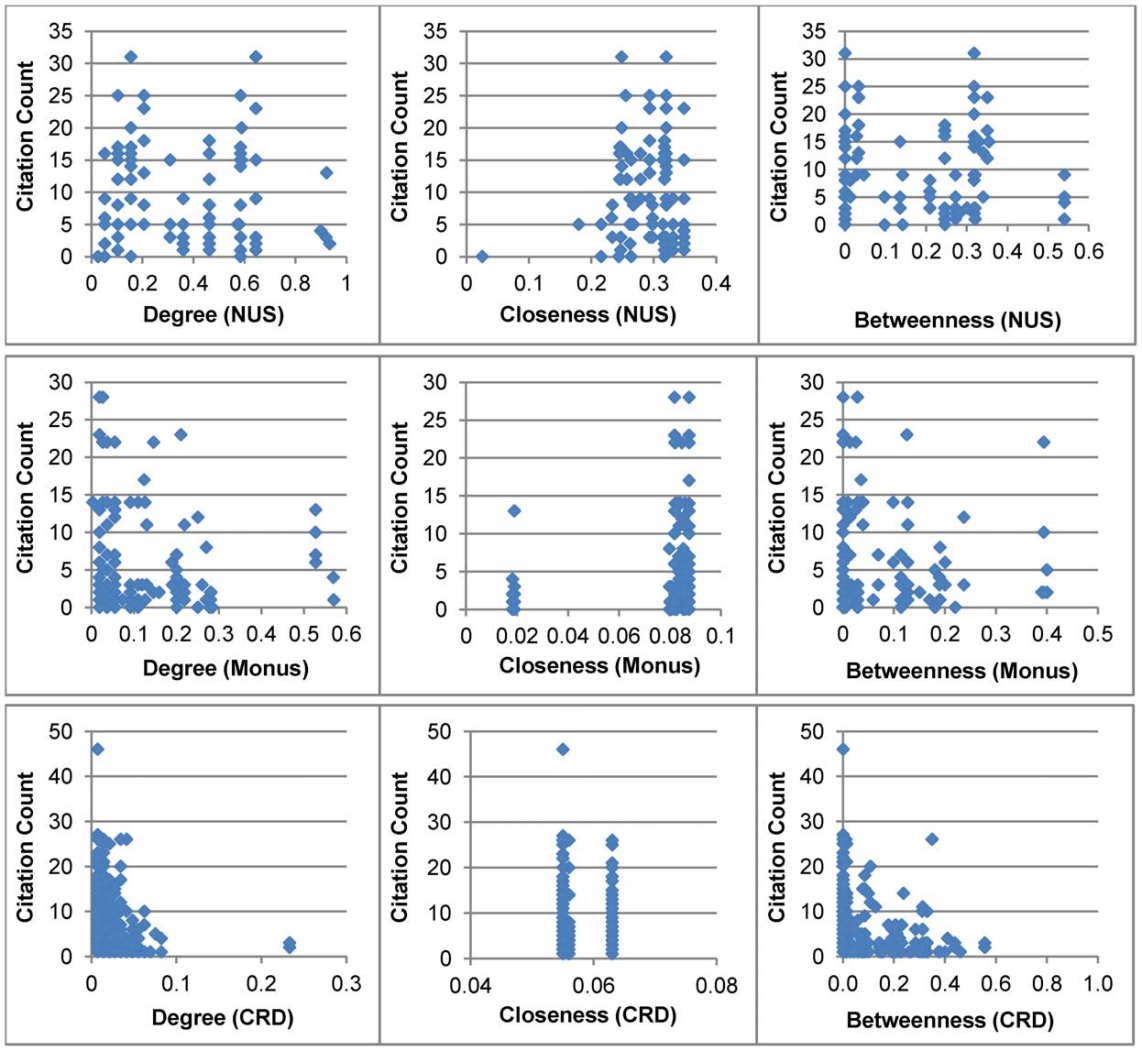
Network attributes for each author and the corresponding *citation count* of the paper. Three basic centrality measures (i.e., *degree centrality*, *closeness centrality* and *betweenness centrality*) are considered. CRD stands for ‘*Complete Research Dataset*’.

We plot the *citation count* of each paper against the network attribute of each of its all co-authors in Figure 4. This figure illustrates how the research dataset look like in terms of network position of each co-author and the corresponding *citation count* of the paper. We considered *degree centrality*, *closeness centrality* and *betweenness centrality* to measure network position of each co-author. A significant difference in *citation counts* of published papers (for the complete research dataset as well as for both NUS and Monus University groups) is noticed for authors who have same values for network measures. This could be explained by the fact that there are few highly connected and well cited authors (e.g., professor) in all three networks and less prominent authors (i.e., less connected and less cited) have co-authored with them (e.g., student-professor link). It is also noticed that *betweenness centrality* values for many authors are zero. These authors could be either students and/or new comers to the scientific field. For this reason, they do not play any bridging role in the co-authorship network.

For the group level research dataset, we then present the top-10 papers in respect of the average values of *degree centrality* and *betweenness centrality*, and their citation counts for both NUS and Monash University research groups in Table 4. Out of ten, eight of them have positions, though in a different rank, in the top-10 lists of *degree centrality* and *betweenness centrality* for NUS. Papers with ID *1649* and *1140* have positions only in the top-10 list of *degree centrality*. Like this, papers with ID *1653* and *1897* have positions only in the top-10 list of *betweenness centrality*. For the Monash University research group, we notice that nine papers (out of ten) have positions, although not in same order, in the top-10 lists of *degree centrality* and *betweenness centrality*. Opposite to *Paper ID 763*, *Paper ID 1810* has position in the top-10 list of *degree centrality* but does not have position in the top-10 list of *betweenness centrality*. Although the values for *degree centrality* and *betweenness centrality* are in order (highest to lowest) in Table 4 corresponding citation counts are not. If we found both of them (i.e., either *degree centrality* and citation count, or *betweenness centrality* and citation count) follow similar ordering (e.g., highest to lowest) from our research dataset then the corresponding correlation coefficient values of Table 3 must be 1.0 (i.e., perfect correlation). We do not find any correlation coefficient value of 1.0 although they are statistically significant. For this reason, although *degree centrality* and *betweenness centrality* values are in order in Table 4 corresponding citation counts do not follow the similar ordering.

**Table 4:** Top-10 papers (in respect of average of *degree centrality* and *betweenness centrality* values of co-authors) and their corresponding citation counts.

	Top-10 papers in respect of centrality measures and their citation counts					
	In respect of *Degree Centrality*			In respect of *Betweenness Centrality*		
	*Paper ID.*	*Degree centrality*	*Citation Count*	*Paper ID.*	*Betweenness centrality*	*Citation Count*
**NUS**	327	0.92	22	327	0.54	22
	1131	0.92	18	1131	0.54	18
	2871	0.64	13	2209	0.35	14
	2876	0.64	12	2871	0.34	13
	2800	0.64	11	2876	0.34	12
	1649	0.58	20	2800	0.34	11
	1140	0.58	18	1653	0.32	15
	983	0.58	13	1897	0.32	9
	1571	0.58	23	1571	0.30	23
	2209	0.58	14	983	0.28	13
**Monash University**	526	0.57	13	1729	0.40	17
	1655	0.53	28	1655	0.39	28
	1729	0.53	17	1137	0.22	28
	437	0.28	14	526	0.20	23
	1137	0.27	23	1128	0.19	16
	1344	0.27	14	437	0.19	15
	1482	0.27	11	1174	0.19	15
	1174	0.26	22	1344	0.18	13
	1128	0.26	14	763	0.17	11
	1810	0.25	13	1482	0.15	10

*Paper ID* is generated by the database system where we keep our research dataset.

### Impact of Network Positions of Authors on Their Strength of Scientific Collaboration

The correlation coefficient values between network centrality of authors and the strength of their scientific collaborations are being presented in Table 5. For the complete research dataset, it is evident that *degree centralities* and *betweenness centralities* of a pair of authors have positive impact on the strength of their research collaboration (rho = 0.331, p<0.01 at 2-tailed and 0.327, p<0.01 at 2-tailed respectively). For the group level research dataset, it is evident that *degree centrality* and *betweenness centrality* of authors in a co-authorship network have an impact on their strength of scientific collaborations for both NUS and Monash University research groups. It is highly expected to have strong *tie strength* between two authors if they have higher *degree centrality*, or *betweenness centrality*, or both. *Closeness centralities* of authors are positively correlated with the strength of their scientific collaboration for NUS but not for Monash University research group and for the complete research dataset.

**Table 5:** Correlation matrix between three network centrality measures and strength of scientific collaboration (i.e., *tie strength*) between two authors.

Correlations					
		Tie Strength (i.e., Strength of scientific collaboration)		Complete research dataset	
Spearman’s rho		NUS	Monash University		
Degree centrality	Correlation Coefficient	.503**	.356**	.331**	
	Sig. (2-tailed)	.001	.005	.001	
	N	101	154	2593	
Closeness centrality	Correlation Coefficient	.278*	.053	.009	
	Sig. (2-tailed)	.045	.259	.199	
	N	101	154	2593	
Betweenness centrality	Correlation Coefficient	.499**	.347**	.327**	
	Sig. (2-tailed)	.019	.007	.001	
	N	101	154	2593	

** Correlation is significant at the 0.01 level (2-tailed).

* Correlation is significant at the 0.05 level (2-tailed).

**Figure 5:**
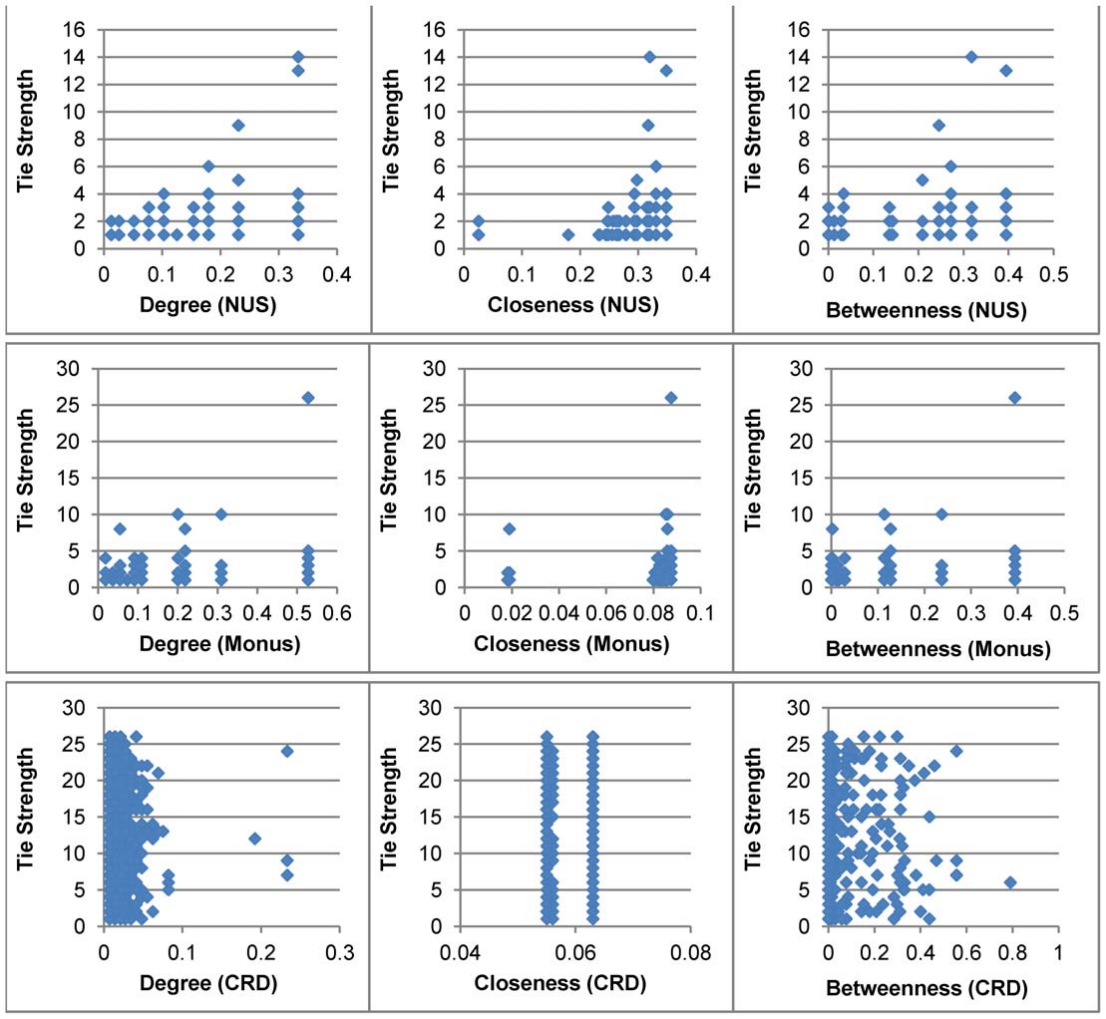
Network attributes for each author and the *tie strength* of that author with all her/his co-authors. Three basic centrality measures (i.e., *degree centrality*, *closeness centrality* and *betweenness centrality*) are considered. CRD stands for ‘*Complete Research Dataset*’.

We then plot the *tie strength* between two co-authors against the network attributes of each of the co-authors in Figure 5. This figure illustrates the research dataset in terms of network position of each author and the *tie strength* with all her/his co-author(s). We considered *degree centrality*, *closeness centrality* and *betweenness centrality* to measure network position of each author. From this figure, it is evident that there is a significant difference in network measures for two co-authors who either form a strong tie or weak tie. This could be explained by a student-supervisor relation where the student, who does not collaborate with any other author, publish many paper (i.e., strong *tie strength*) or very few paper (i.e., low *tie strength*) with the supervisor who is highly connected and well cited. Some of the authors do not play any bridging role in the co-authorship network as reflected in the *betweenness centrality* values (some of these values are zero).

Table 6 presents the top-10 collaborations among authors in respect of *tie strength* for the group level research dataset. The highest *tie strength* value for NUS research group is 3 whereas for Monash University research group this value is 4. For some authors from both NUS and Monash University research groups, it is evident that they have a high *degree centrality* but low *betweenness centrality*. It could be explained by the fact that some authors have high number of collaborations with many different other authors; however, they do not play a bridging role in the co-authorship network. For this reason, they have high *degree centrality* but low *betweenness centrality*. In similar way, the presence of some authors in our dataset having low *degree centrality* and high *betweenness centrality* could be interpreted.

**Table 6:** Top-10 collaborations between authors (in respect of *tie strength*) and scaled network measures (i.e., *degree centrality* and *betweenness centrality*) of corresponding collaborators.

Top-10 collaborations in respect of tie strength								
	*No.*	*Author 1*			*Author 2*			*Tie Strength*
		*Name*	*Degree*	*Betweenness*	*Name*	*Degree*	*Betweenness*	
**NUS**	1	N.E. Shanmugam	0.58	0.35	J.Y.R. Liew	0.92	0.54	3
	2	N.E. Shanmugam	0.58	0.35	J.Y.R. Liew	0.92	0.54	3
	3	H. Chen	0.16	0.23	J.Y.R. Liew	0.92	0.54	3
	4	S.L. Lee	0.34	0.12	J.Y.R. Liew	0.92	0.54	3
	5	L.K. Tang	0.16	0.03	J.Y.R. Liew	0.92	0.54	3
	6	C.H. Yu	0.21	0.23	J.Y.R. Liew	0.92	0.54	2
	7	K.M.A. Sohel	0.08	0.03	J.Y.R. Liew	0.92	0.54	2
	8	M. Dhanalakshmi	0.05	0.03	N.E. Shanmugam	0.58	0.35	2
	9	C.Y. Liaw	0.13	0.01	T. Balendra	0.26	0.24	2
	10	Y.S. Choo	0.13	0.01	J.Y.R. Liew	0.92	0.54	2
**Monash University**	1	M.R. Bambach	0.11	0.20	H.H. Jama	0.11	0.04	4
	2	L.H. Han	0.09	0.02	X.L. Zhao	0.53	0.39	4
	3	H. Jiao	0.01	0.02	X.L. Zhao	0.53	0.39	4
	4	J. A. Packer	0.06	0.01	X. Zho	0.31	0.24	3
	5	D. V. Binh	0.09	0.05	X.L. Zhao	0.53	0.39	3
	6	D. V. Binh	0.09	0.05	R. Al-Mehaidi	0.22	0.13	3
	7	Z. Tao	0.10	0.01	X.L. Zhao	0.53	0.39	3
	8	T. Kitada	0.16	0.11	M. Elchalakani	0.05	0.03	3
	9	R.H. Grzebieta	0.09	0.02	H.H. Jama	0.11	0.04	2
	10	R.H. Grzebieta	0.09	0.02	M.R. Bambach	0.11	0.20	2

### Regression Models for Citation Count and Tie Strength

We developed regression models for *citation count* and *tie strength* using the complete research dataset as well as NUS and Monash University data. These models are summarized in Table 7. All the beta values of this table are significant. That means, *degree centrality* and *betweenness centrality* have a significant impact on the *citation count* of a scientific publication and *tie strength* between two authors. Both *citation count* and *tie strength* can be measured from the corresponding *degree centrality* and *betweenness centrality* values. For example, the relations among *degree centrality*, *betweenness centrality*, and citation count for NUS dataset can be represented by the following equation:
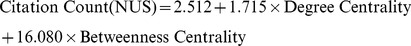



**Table 7:** Regression models for Complete research dataset, and NUS and Monash University dataset.

Model	Dependent Variable	R^2^ Value	Constant	Independent Variable/Predictors	β	Significance
1 Complete research dataset	Citation Count	0.512	1.245	Degree Centrality	2.913	.002
				Betweenness Centrality	8.381	.001
2 Complete research dataset	Tie Strength	0.591	2.128	Degree Centrality	1.341	.003
				Betweenness Centrality	7.839	.001
3 (NUS)	Citation Count	0.633	2.512	Degree Centrality	1.715	.000
				Betweenness Centrality	16.080	.001
4 (NUS)	Tie Strength	0.559	0.107	Degree Centrality	2.306	.002
				Betweenness Centrality	9.712	.001
5 (Monash)	Citation Count	0.542	3.243	Degree Centrality	5.977	.002
				Betweenness Centrality	12.946	.001
6 (Monash)	Tie Strength	0.618	0.998	Degree Centrality	3.414	.001
				Betweenness Centrality	19.215	.000

## Discussion and Conclusion

This research is motivated by two research questions: (i) “how is the citation a count of a scientific paper influenced by the network positions of its co-author(s) in a co-authorship network?” and (ii) “how is the strength of relations between two authors influenced by their network positions in a co-authorship network?” In answer to these research questions, we observe that citation count of a scientific paper is affected by the degree centrality and betweenness centrality of its co-author(s). We also find that degree centrality and betweenness centrality of a pair of authors have positive impact on their strength of scientific collaboration in a co-authorship network. The corresponding correlation coefficient values for degree centrality and betweenness centrality are ranging from 0.326 to 0.503. All of these values are statistically significant although they are not showing perfect or upper level correlation (i.e., correlation coefficient is close to 1). A small correlation coefficient value could be statistically significant if sample size is high; whereas, for a small sample size (e.g., 35) a high correlation coefficient value would not be statistically significant [38]. A correlation coefficient value of 0.04, for instance, would be statistically significant for a sample size of 10,000 [38].

For ordinary social networks (e.g., friendship network), it has earlier been shown that strong ties are associated with dense network neighbourhoods while weaker ties act as bridges [34], [39]. Because of their bridging capability, weak ties are considered as bottlenecks for the diffusion of information. However, Pan and Saramäki [40] show that dense local neighbourhoods mainly consist of weak ties and strong ties are more important for overall connectivity in a co-authorship network. This is because the strong ties (e.g., between professors) are there for longer time as compared weak ties (e.g., between student-professor). In this study, we find that, in a co-authorship network, strong ties between authors are associated with network centralities of *degree* and *betweenness*. That means, strong ties are associated with dense network neighbourhoods. Therefore, unlike Pan and Saramäki [40], the findings of this study are in align with many other earlier studies (e.g., [34]) on ordinary social networks. The difference in the findings between our study and Pan and Saramäki [40] could be explained by the fact that the evolutionary patterns of co-authorship networks are not similar in different research contexts [41]. This study utilizes dataset from ‘*steel structure*’ research area; whereas, they used archive dataset that contains publication from different domains.

A research paper can attract high volume of citation when it facilitates knowledge creation and innovation [42], [43]. Thus, the findings (related to *citation count*) of this study elicit positional characteristics of prolific authors, in terms of knowledge and innovation, in a co-authorship network. According to our finding, more frequently cited papers are mostly co-authored by scientists who have higher *degree centrality* and *betweenness centrality* in a co-authorship network. That indicates authors, who have more connectivity (i.e., *degree centrality*) and capacity to control the flow of information (i.e., *betweenness centrality*), are contributing more to knowledge creation and innovation compared to other authors, who have less connectivity and less information control in a co-authorship network.

This research is not without its limitations. This research was conducted using co-authorship dataset for only two research groups from a single research discipline (i.e., *‘steel structures’*). Hence, studies involving datasets from more research groups and research areas as well as from inter-disciplinary research areas are needed before we can arrive at more definitive conclusions regarding the generic nature of our research findings.

As evidenced in the current co-authorship literature, most of the research on co-authorship network analysis focus on the overall topology of networks, analysis of statistical properties of individuals, and relationship between citation and centrality measures at author level. However, to our knowledge, there is no such study in the literature that seeks the impact of network positions of authors in a co-authorship network on the citation counts of scientific publications and the *tie strength* of scientific collaborations between authors.

## References

[1] Eom YH, Fortunato S (2011) Characterizing and modeling citation dynamics. PloS one 6: e24926. 21966387 10.1371/journal.pone.0024926 PMC3178574

[2] Wallace ML, Larivière V, Gingras Y (2012) A small world of citations? The influence of collaboration networks on citation practices. PloS one 7: e33339. 22413016 10.1371/journal.pone.0033339 PMC3296690

[3] Gingras Y, Larivière V, Macaluso B, Robitaille JP (2008) The effects of aging on researchers’ publication and citation patterns. PloS one 3: e4048. 19112502 10.1371/journal.pone.0004048 PMC2603321

[4] Scott J (2005) Social network analysis: A handbook. London: Sage Publications Ltd.

[5] Newman MEJ (2001) The structure of scientific collaboration networks. Proceedings of the National Academy of Sciences of the United States of America 98: 404. 11149952 10.1073/pnas.021544898 PMC14598

[6] Uddin S, Hossain L, Abbasi A, Rasmussen K (2012) Trend and efficiency analysis of co-authorship network. Scientometrics 90: 687–699.

[7] Abbasi A, Hossain L, Uddin S, Rasmussen KJR (2011) Evolutionary dynamics of scientific collaboration networks: multi-levels and cross-time analysis. Scientometrics 89: 687–710.

[8] Radicchi F, Fortunato S, Vespignani A (2012) Citation networks. Models of Science Dynamics. 233–257.

[9] Oldenburg H (1665) Epistle Dedicatory. Philosophical Transactions of the Royal Society of London 1.

[10] Petit M (1665) A Relation of the Advice Given by Monsieur Petit, Intendant of the Fortifications of Normandy. Touching the Conjunction of the Ocean and Mediterranean. Philosophical Transactions 1: 41–43.

[11] Moray R, Du Son M (1665) A Way to Break Easily and Speedily the Hardest Rocks, Communicated by the Same Person, as He Received It from Monsieur Du Son, the Inventor. Philosophical Transactions 1: 82–85.

[12] Luukkonen T, Tijssen RJW, Persson O, Sivertsen G (1993) The measurement of international scientific collaboration. Scientometrics 28: 15–36.

[13] Bošnjak L, Marušić A (2012) Prescribed practices of authorship: review of codes of ethics from professional bodies and journal guidelines across disciplines. Scientometrics: 1–13.

[14] Liao CH, Yen HR (2012) Quantifying the degree of research collaboration: A comparative study of collaborative measures. Journal of Informetrics 6: 27–33.

[15] Hoekman J, Frenken K, Tijssen RJW (2010) Research collaboration at a distance: Changing spatial patterns of scientific collaboration within Europe. Research Policy 39: 662–673.

[16] Mazloumian A (2012) Predicting Scholars’ Scientific Impact. PloS one 7: e49246. 23185311 10.1371/journal.pone.0049246 PMC3504022

[17] Mazloumian A, Eom YH, Helbing D, Lozano S, Fortunato S (2011) How citation boosts promote scientific paradigm shifts and nobel prizes. PloS one 6: e18975. 21573229 10.1371/journal.pone.0018975 PMC3087729

[18] Kretschmer H (1994) Coauthorship networks of invisible colleges and institutionalized communities. Scientometrics 30: 363–369.

[19] Persson O, Beckmann M (1995) Locating the network of interacting authors in scientific specialties. Scientometrics 33: 351–366.

[20] Melin G, Persson O (1996) Studying research collaboration using co-authorships. Scientometrics 36: 363–377.

[21] Hoffman P (1987) The man who loves only numbers. Atlantic Monthly 260: 60–74.

[22] Inzelt A, Schubert A, Schubert M (2009) Incremental citation impact due to international co-authorship in Hungarian higher education institutions. Scientometrics 78: 37–43.

[23] Cho CC, Hu MW, Liu MC (2010) Improvements in productivity based on co-authorship: a case study of published articles in China. Scientometrics 85: 463–470.

[24] Luukkonen T, Persson O, Sivertsen G (1992) Understanding patterns of international scientific collaboration. Science, Technology & Human Values 17: 101.

[25] Newman MEJ (2004) Coauthorship networks and patterns of scientific collaboration. Proceedings of the National Academy of Sciences of the United States of America 101: 5200–5205. 14745042 10.1073/pnas.0307545100 PMC387296

[26] Liu X, Bollen J, Nelson ML, Van de Sompel H (2005) Co-authorship networks in the digital library research community. Information Processing & Management 41: 1462–1480.

[27] Yan E, Ding Y (2009) Applying centrality measures to impact analysis: A coauthorship network analysis. Journal of the American Society for Information Science and Technology 60: 2107–2118.

[28] Meho LI, Yang K (2007) Impact of data sources on citation counts and rankings of LIS faculty: Web of Science versus Scopus and Google Scholar. Journal of the American Society for Information Science and Technology 58: 2105–2125.

[29] Wasserman S, Faust K (2003) Social network analysis: Methods and applications. Cambridge: Cambridge University Press.

[30] Uddin S, Murshed STH, Hossain L (2011) Power-law behaviour in complex organizational communication network during crisis. Physica A: Statistical Mechanics and its Applications 390: 2845–2853.

[31] Uddin MS, Hossain L (2011) Social Networks Enabled Coordination Model for Cost Management of Patient Hospital Admissions. Journal for Healthcare Quality 33: 37–48. 10.1111/j.1945-1474.2011.00118.x 23845132

[32] Hamra J, Uddin S, Hossain L (2011) Exponential random graph modeling of communication networks to understand organizational crisis. New York. ACM. 71–78.

[33] Uddin S, Hossain L, Murshed ST, Crawford JW (2011) Static versus dynamic topology of complex communications network during organizational crisis. Complexity 16: 27–36.

[34] Granovetter M (1973) The strength of weak ties. American journal of sociology 78: 1360–1380.

[35] Bavelas A (1950) Communication patterns in task-oriented groups. Journal of the acoustical society of America 22: 725–730.

[36] Freeman L (1978) Centrality in social networks: Conceptual clarification. Social Networks 1: 215–239.

[37] Carley K (2010) Center for Computational Analysis of Social and Organizational Systems (CASOS), Institute for Software Research International (ISRI). School of Computer Science, Carnegie Mellon University, 5000 Forbes Avenue Pittsburgh, PA 15213–3890.

[38] Field A (2009) Discovering statistics using SPSS: Sage Publications Ltd.

[39] Granovetter M (1983) The strength of weak ties: A network theory revisited. Sociological theory 1: 201–233.

[40] Pan RK, Saramäki J (2012) The strength of strong ties in scientific collaboration networks. EPL (Europhysics Letters) 97: 1–6.

[41] Acedo FJ, Barroso C, Casanueva C, Galán JL (2006) Co-Authorship in Management and Organizational Studies: An Empirical and Network Analysis. Journal of Management Studies 43: 957–983.

[42] Hu MC (2011) Evolution of knowledge creation and diffusion: the revisit of Taiwan’s Hsinchu Science Park. Scientometrics 88: 949–977.

[43] Zou G, Yilmaz L (2011) Dynamics of knowledge creation in global participatory science communities: open innovation communities from a network perspective. Computational & Mathematical Organization Theory 17: 35–58.

